# Supplementary material for: Layered metals as polarized transparent conductors
Source: Nat Commun. 2023 May 30;14:3147. doi: 10.1038/s41467-023-38848-0 (PMC10229626; doi:10.1038/s41467-023-38848-0)
Supplement: Supplementary file 1 — Supplementary Information [file 41467_2023_38848_MOESM1_ESM.pdf]

Supplementary Information

Characterization of Tl2201 microstructures

The in-plane resistivity of Tl2201 has been characterized in micro-structured current path of bulk single crystals. A lamella approach as taken for SRO was not possible as the amorphous surface layer in Tl2201 does not promote ohmic contacts. The crystals were contacted with Dupont 4929 silver paint and sputtered gold (150nm thick) which was subsequently annealed at 400°C for 15 minutes.

Electrical resistivity measurements were carried out using a Quantum Design Physical Property measurement system (PPMS).

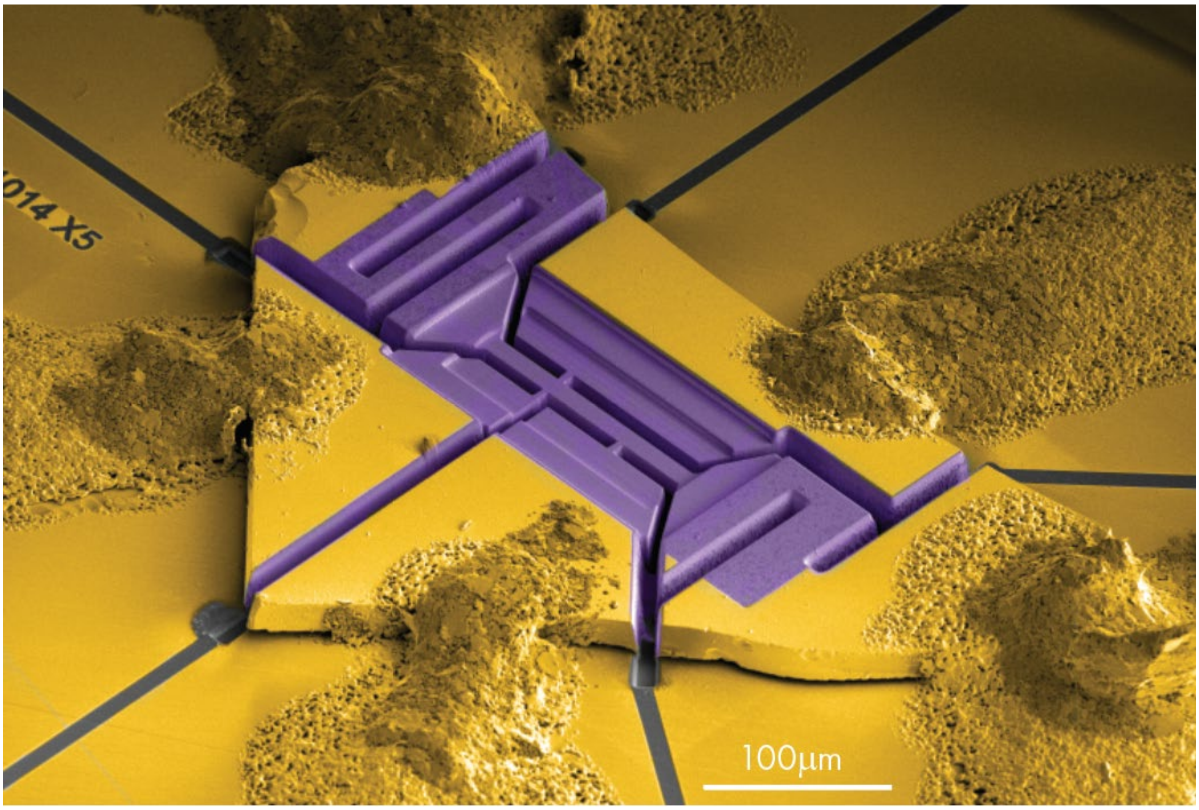

Supplementary Figure 1: Tl2201 microstructure. Scanning electron microscope image of a microstructured bulk single crystal for of Tl2201 for in-plane electrical resistivity measurements. Meander are cut on each side of the current path to avoid c-axis contribution to the resistivity.

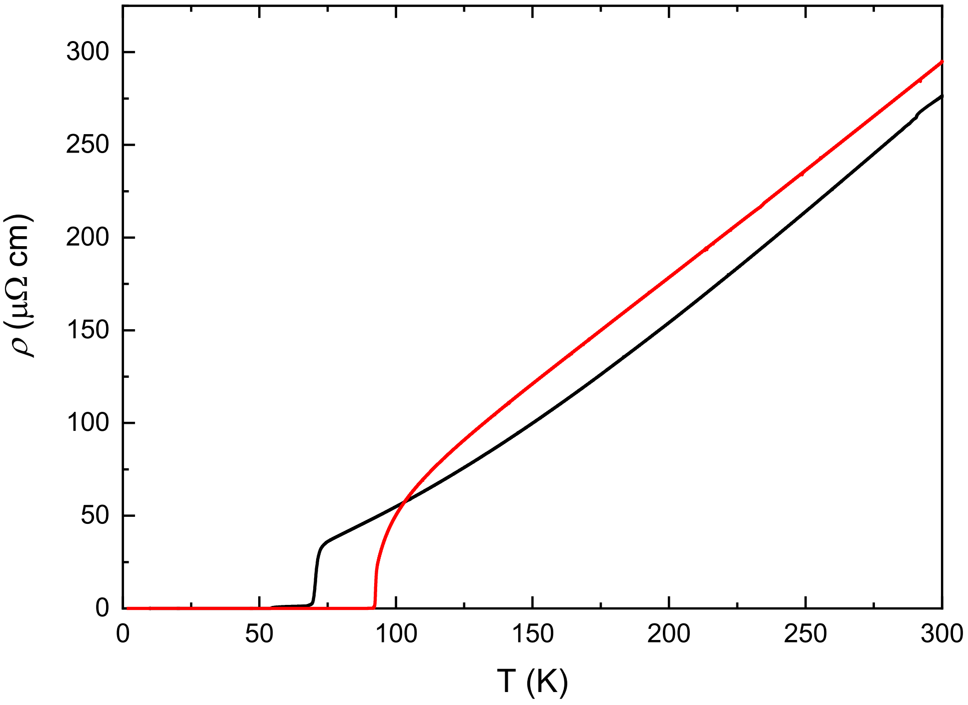

Supplementary Figure 2: Resistivity of Tl2201 microstructures. The data shows the in-plane resistivity of optimally doped and overdoped Tl2201 microstructures. The measured resistivity is in good agreement with that obtained on bulk single crystals[15,43].

Analysis of Commercially available linear Polarizer

For the market analysis of commercial polarizer we use the parameters studied on SRO in this work as evaluation criteria. Those are the transmission in the visible range and extinction ratio maximum.

Visible Wire Grid Polarizer. Here we use the Thorlabs WP12L-VIS for comparison. The datasheet shows a Transmission of over 90% over most of the visible range. The high extinction ratio of over 1000 (up to 1800) is strongly wavelength dependent.

Ultra Broadband Wire Grid Polarizer. The system used for comparison is the Thorlabs WP12L-UB. The Polarizer shows a transmission of over 70% for wavelength of 400 to 2600nm with >80% for wavelengths above 1000nm. The polarizer shows a high extinction ration of more than 10000, which similar to the visible range product is strong frequency dependent. The highest values of more than 30000 are achieved in the long wave length limit and require fine wavelengths of the used laser, due to the narrow bandwidth of the high extinction ration.

Nano Particle Linear Film Polarizer. Here we chose two different products the LPUV-series and LPVISA-series, for UV and the optical spectra respectively. Both systems show extremely high extinction ratios of more than 10^6. Looking at the wavelength dependence the extinction ratio rapidly decreases beyond the

proposed use range and drops below that shown for SRO. For LPVISA this already happens at wavelengths of 600nm. Another downside of these systems is the low and strongly wavelength dependent transmission which lies below 60% in the UV and ranges from 60% to 80% in the visible system.

In summary, while commercially available polarizers are characterized with high extinction ratios of more than 10^6 and transmission ratios of over 90%, they are often most useful in carefully tuned systems due to the peak performance in a narrow bandwidth. None of the above systems show a similarly wavelength independent extinction ratio and optical transmission as the SRO results presented in this work. The application of SRO as polarizer poses the opportunity for less complex setups when using broad band light sources.

Analysis of Crystallinity

In order to establish that the transparent matter is of single crystal form we have used X-ray diffraction using a Rigaku Synergy-S diffractometer.

The spot size for the X-ray diffraction is on the order of 100μm and fits within a silicon nitride membrane on which a microstructured sample of Sr_2RuO_4 was mounted (Fig S3). By integration over images taken at different scattering angle we have obtained the pattern shown in Fig S4. The rings seen in the image are also visible away from the sample and stem from the non-stoichiometric SiN-membrane. A subset of the reflections stem from the Si-frame which holds the sample. In addition to those peakes we identified the majority of reflection to stem from a crystal structure of class I4/m with a = 3.86 Å, b = 3.91 Å and c = 12.7 Å. This is in good agreement with the expected crystal structure and lattice parameter of Sr_2RuO_4 [44]. This demonstrates that the transparent material retains its bulk crystal structure.

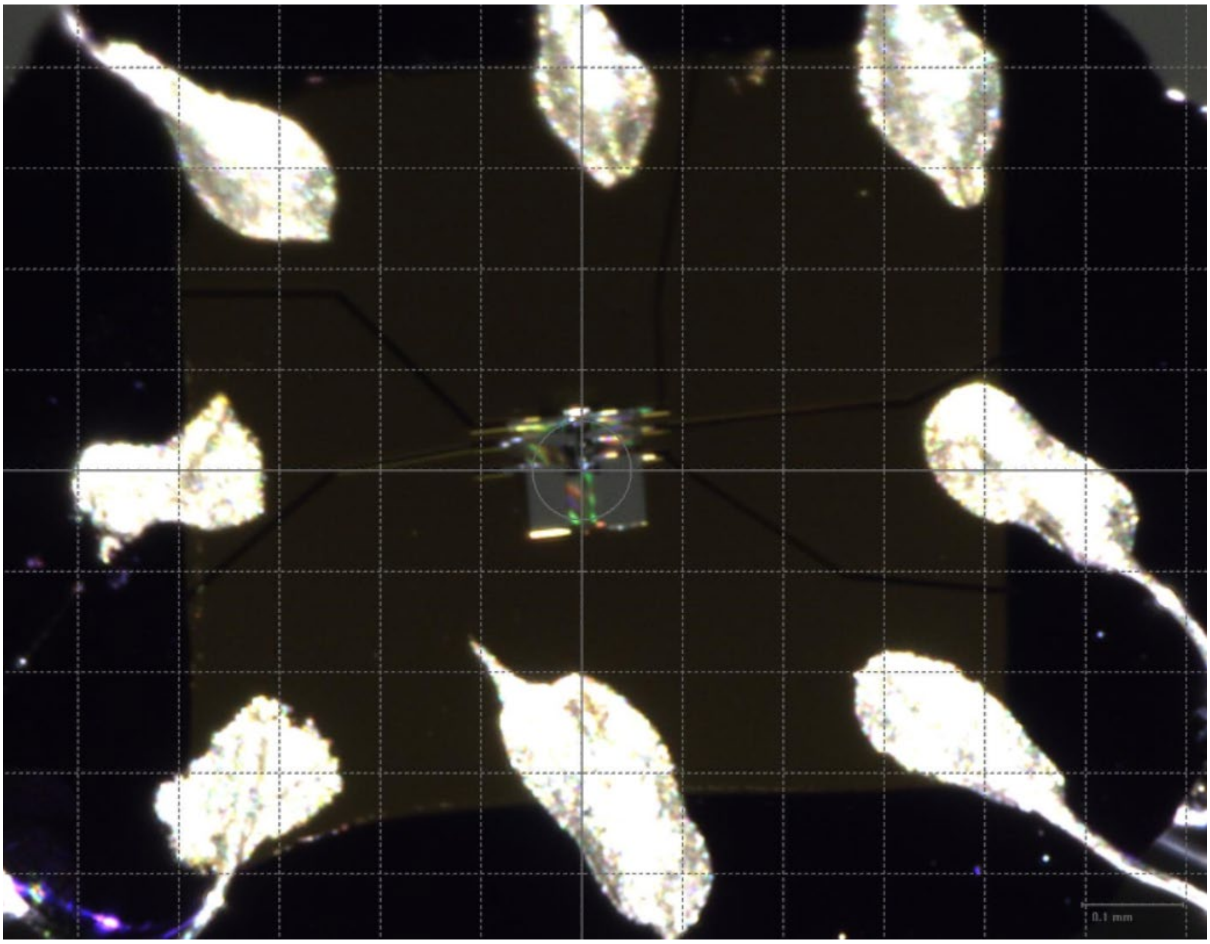

Supplementary Figure 3: Optical image of the XRD experiment. Image of the alignment for transmission X-ray diffraction. The circle represents the XRD beam size and position. It was adjusted to lay in the 100x100µm window of the SiN-membrane. Using a monochromatic Mo-source we obtain enough signal strength to determine the lattice parameter of the microscture.

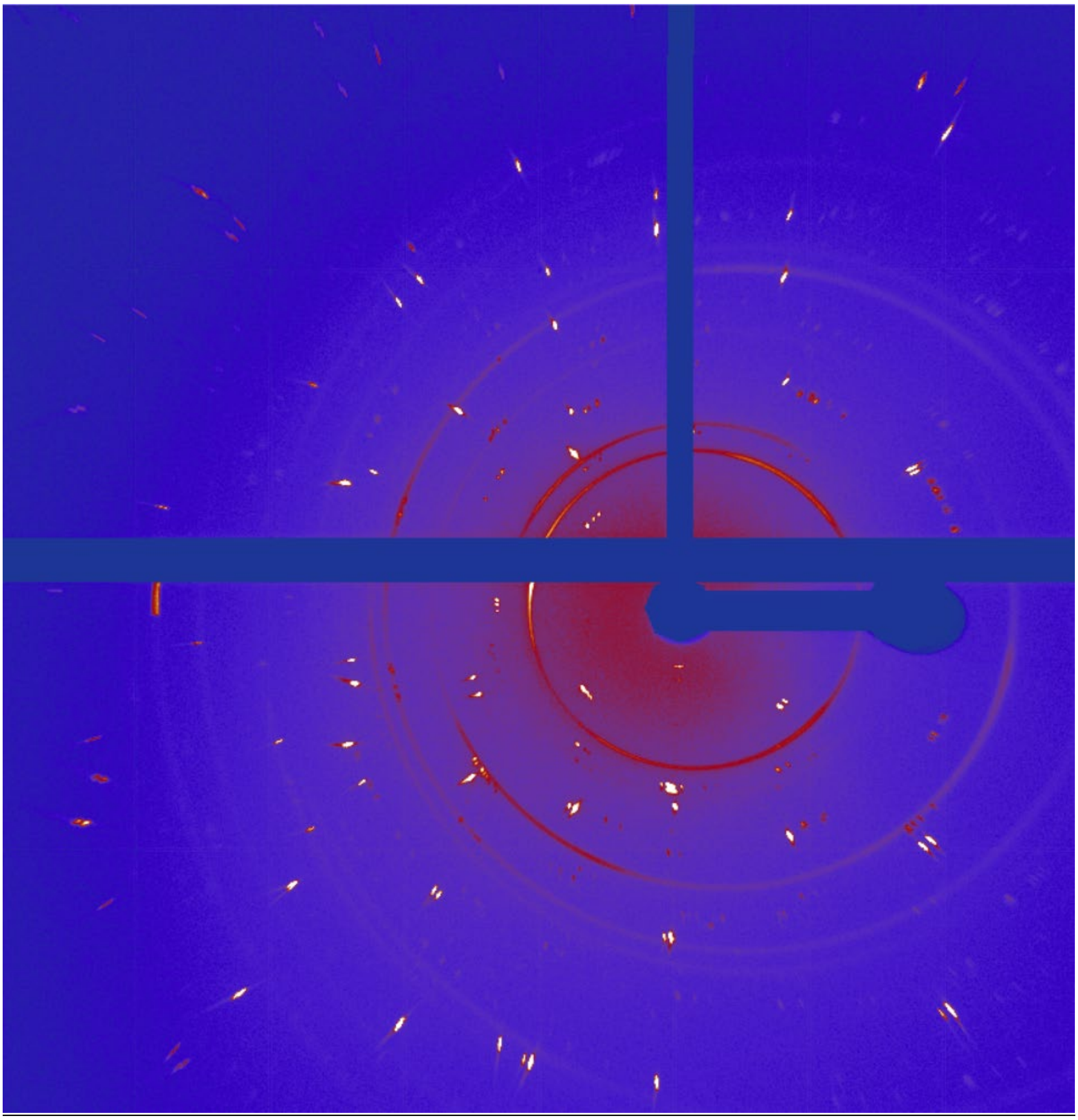

Supplementary Figure 4: X-ray diffraction image of microstructure SRO sample. Integrated reflection image of transmission XRD configuration of SRO microstructure.
